# #HowNotToDoPatientEngagement: the engaging with purpose patient engagement framework based on a twitter analysis of community perspectives on patient engagement

**DOI:** 10.1186/s40900-023-00527-1

**Published:** 2023-12-13

**Authors:** Brianna Dunstan, Francine Buchanan, Alies Maybee, Aisha Lofters, Ambreen Sayani

**Affiliations:** 1 Women’s College Research Institute, Women’s College Hospital, 76 Grenville St, Toronto, ON M5S 1B2 Canada; 2 Patient and Family Advisor, Toronto, Canada; 3 Department of Family and Community Medicine, University of Toronto, Toronto, ON Canada; 4 Institute of Health Policy, Management and Evaluation, University of Toronto, Toronto, ON Canada

**Keywords:** Patient engagement, Patient perspective, Patient advisory, Framework, Patient engagement framework

## Abstract

**Background:**

Evaluation of patient engagement practices are frequently researcher-driven, researcher-funded, and asymmetric in power dynamics. Little to no literature on patient experiences in patient engagement exist that is are not framed by institutionally-driven research inquiries (i.e., from the lens of a research team lead, or healthcare administrative setting). Understanding these perspectives can help us understand: (i)what matters to patients when they are engaged in research; (ii)why it matters to them, and(iii) how to improve patient engagement practices, so that the needs and priorities of patients are consistently met.

**Methods:**

This is a patient partner-initiated study. Study authors (including patient partners) conducted a conventional and summative content analysis of textual data retrieved from a highly engaged conversation on Twitter regarding the hashtags #HowNotToDoPatientEngagement and #HowToDoPatientEngagement posted between February 2018 to June 2021. Twitter is a microblogging platform that allows for free-flowing discussions between users not pre-bound by specific community groupings (like within that of Facebook).

**Results:**

A total of 276 tweets were retrieved from 178 separate contributors across seven geographical locations. Four stakeholder groups were identified. We generated 24 codes, nine subthemes and five overarching themes: respect, support, collaboration, inclusivity and impact. Four of these themes are closely aligned with the Strategy for Patient Oriented (SPOR) Patient Engagement framework. We identify impact as a separate and new theme.

**Interpretation:**

Based on our findings we offer the Engaging with Purpose Patient Engagement Framework that defines and describes respect, support, collaboration, inclusivity and impact as five key pillars of meaningful patient engagement.

## Plain Language Summary

Patient engagement in research is an important aspect of healthcare, but most studies on the topic are driven by researchers and healthcare settings. We wanted to understand patient experiences of patient engagement from the lens of patient partners that occurred outside the traditional norms of evaluating such engagements. To do this, we analyzed data from Twitter, a platform where users freely share their thoughts and opinions. Posts that included the hashtags #HowNotToDoPatientEngagement and #HowToDoPatientEngagement were of focus because they resonated with members of the online patient partner community who wanted to improve the current state of patient partnership. We identified 276 tweets from 178 contributors across seven geographic locations. Five themes emerged from the study: respect, support, collaboration, inclusivity, and impact which led to the development of the Engaging with Purpose Patient Engagement Framework. This framework highlights respect, support, collaboration, inclusivity, and impact as the five essential pillars of meaningful patient engagement in research. By understanding and implementing these pillars, researchers and institutions can improve patient engagement practices and ensure that the needs and priorities of patients are effectively addressed.

## Introduction

Patient engagement (PE) is an approach to the design, planning and research of health services that promotes active and meaningful collaboration between persons with lived/living-experience and partners in the healthcare system [1–3]. When done well, patient engagement ensures that the patient is not a “passive receptor” of health interventions, but rather is a “proactive partner” who is able to shape the existing healthcare structure and its outcomes. However, to ensure engagement is done well, it is important to understand the perspectives of all parties involved. The aim of this patient partner-initiated study was to investigate patient engagement through the analysis of a pre-existing online patient engagement community organically formed through Twitter application users. This community rallied around the hashtag, #HowNotToDoPatientEngagement and consisted of a subset of online Twitter patient partner community members and others who engage with patient partners in this shared dialogue. The hashtag provided a unique opportunity to analyse self-reported perspectives on the practice of patient engagement within healthcare, understand what matters to patients when they are engaged in research, why it matters to them, and offers insights on how to improve the PE process so that the needs and priorities of patients are consistently met.

The Canadian Institutes of Health Research (CIHR) and their Strategy for Patient-Oriented Research (SPOR) launched in 2011[3], and has been instrumental in supporting the growth of patient engagement in Canada, evident by the exponential increase in SPOR research investments [4]; the growing number of hospital research advisory committees to support POR [5]; and the burgeoning literature available to guide PE [6, 7]. However, research to support PE practices is frequently based out of institutions (hospitals and/or research centres) and bound within the time and resource confines of a research study [8]. This structure extends itself to the evaluation of PE, which is frequently researcher-driven, researcher-funded and inherently unbalanced in power dynamics. Little to almost no literature exists that documents the patient partner’s experience in PE outside of this setting. Our study aimed to highlight and explore the perspectives posted on Twitter that were not limited by a specific research scope, thus allowing contributors to provide personal reflections and opinions on aspects of PE that were important to them without specific prompting or framing. This Twitter conversation and our resulting analysis, illuminates current attitudes towards PE, and findings that reflect areas for growth in PE practices.

## Methods

To collect the perspectives of the community that resonated with this online conversation, the Twitter application programming interface Tweet Binder was used for data collection. Tweet Binder conducted a historical report for the searchable terms ‘#HowToDoPatientEngagement’ (#HTDPE) and ‘#HowNotToDoPatientEngagement’ (#HNTDPE) across Twitter’s database. The search consisted of all tweets with any combination of the search terms, in which the language of the tweets collected was English. The historical report was comprised of tweets posted from the initial tweet of #HNTDPE in February of 2018, to the date of report request submission (June 14, 2021). The historical report data encompassed contributors’ usernames, contributor locations (self-reported), timestamps of tweets posted, tweet content, and permanent links to the tweets collected. Tweet Binder’s historical analysis generated a report consisting of 276 tweets that included the searchable hashtags used either exclusively or together within the same post (i.e., #HTDPE, #HNTDPE, or #HTDPE#HNTDPE/#HNTDPE#HTDPE). Of the 276, 82 tweets were original and 194 were retweets/replies, with a total of 178 contributors to the hashtag conversation of interest.

### Ethics approval

All contributor information was collected from a publicly accessible social media platform, Twitter, and all data used in our analysis was self-reported and knowingly posted on a public domain. The Canadian Tri-Council Policy Statement 2 (TCPS 2) states that information that is “in the public domain and the individuals to whom the information refers [has] no reasonable expectation of privacy” and is exempt from Research Ethic Board (REB) review [9]. This was confirmed by our institutional REB, and we did not seek ethics approval.

### Approach to analysis

**Fig. 1:**
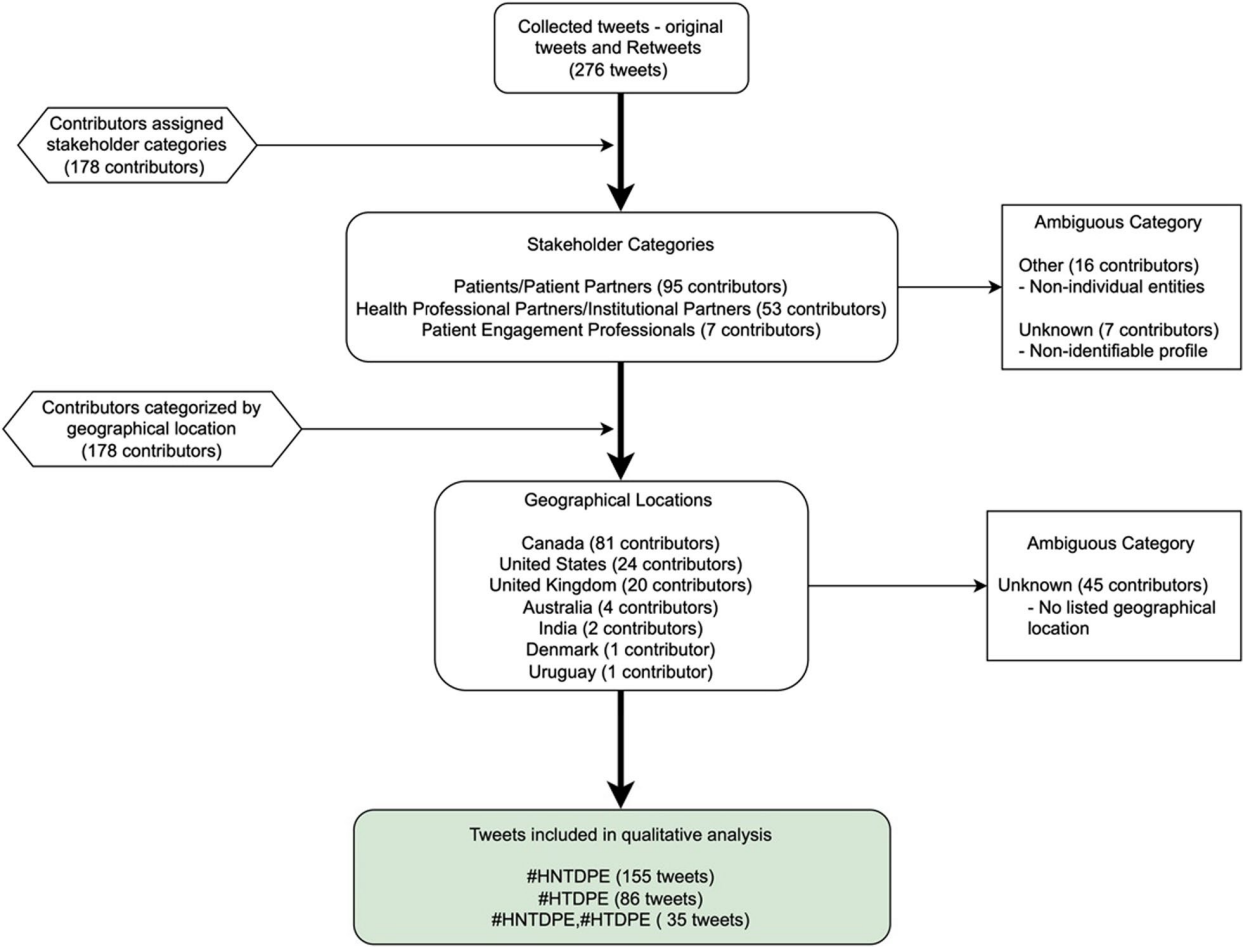
Methodological sorting of raw data

The initial quantitative analysis began by assigning each Twitter contributor into a stakeholder category (Fig. 1). Stakeholders were assigned categories based on their username and Twitter profile biography. Contributors were classified as “Other” if they did not represent an individual entity (i.e., patient advocacy and public health groups). In addition, contributors were classified as “Unknown” if there was too little information in their Twitter biography to accurately assign them into a category. Historical report data was also used to sort contributors by geographical location (Fig. 1).

The data was interpreted using both conventional and summative content analysis [10]. The conventional approach was used to locate codes within the data without the use of preconceived categories. The summative approach was used to identify key words within the text for the purpose of understanding the contextual nature of the hashtag thread. The 276 tweets were read line by line independently by three authors BD, AS and FB. To reduce the burden of analysis, an independent review of a subset of tweets was conducted by patient partner AM, All authors independently took a similar approach to reviewing the tweets, reading and re-reading each tweet, first on its own, then again within the context of the broader #HNTDPE conversation. Emerging codes were discussed amongst all authors, meaning units were created and categorized such that all tweets were mutually exclusive and fit into a single category to avoid over-calculation bias. Finally, a coding framework to guide subsequent coding was agreed upon through group discussions. Two authors independently coded the data (BD, FB) and discrepancies in coding were resolved through group discussion and consensus among all five authors.

## Results

### Twitter contributor’s demographics

A total of 276 tweets posted by 178 contributors were included in the historical report. The international distribution of contributors is as follows: Canada (n = 81), United States (n = 24), United Kingdom (n = 20), Australia (n = 4), India (n = 2), Denmark (n = 1) and Uruguay (n = 1). 81 tweets which originated from Canada were distributed as follows: Ontario (n = 32), British Columbia (n = 12), Nova Scotia (n = 11) and New Brunswick (n = 10).

The Stakeholder categories are based on analysis of each of the 178 contributor’s Twitter profile and include: ‘Patient/Patient Partner’, ‘Health Professional/Institutional Partner’, ‘Patient engagement Professional’, ‘Other’ and ‘Unknown’. The most common contributors comprised of individuals in the ‘Patient/Patient Partner’ stakeholder category (n = 95) followed by ‘Health Professional/Institutional Partners’ that encompassed clinicians, researchers, and health practitioners (c = 53). The proportion of individuals in each stakeholder category that contributed to the overall Twitter conversation can be seen in Table 1.

**Table 1:** Stakeholder categories contributing to Twitter conversation

Stakeholder categories [2]	Count
Patients/patient partners	95
Health professional partner/institutional partner	53
Patient engagement professional	7
Other	16
Unknown	7
Total	178

### Thematic analysis

A total of 276 tweets (n = 276) were coded line-by-line. 12 codes were identified from #HNTDPE, 13 codes from #HTDPE and 4 codes from the combined hashtags of #HTDPE#HNTDPE. In total we generated 29 codes which were then viewed collectively and grouped into subthemes based on similarities, while minimizing overlap between concepts (Table 2).

**Table 2:** Codes by hashtag

#HNTDPE (how NOT to do patient engagement) # of tweets = 155	#HTDPE (how to do patient engagement) # of tweets = 86	#HTDPE#HNTDPE (how to do patient engagement) and (how NOT to do patient engagement) # of tweets = 35
Collaboration	Accessibility	Resources
Crowdsourcing advice	Accreditation/validity’	Promoting events
Disrespected	Appropriate compensation	Promoting Hashtag
Feeling unvalued	Collaboration	Training
Inaccessibility	Crowdsourcing advice	
Inclusion without Impact	Feeling fulfilled	
Lack of/inappropriate compensation	Feeling valued	
Learning from others	Impactful engagement	
Othering	Learning from others	
Promoting Hashtag	Recognizing PE leadership/advisors	
Promoting events	Resources	
Training	Promoting events	
	Training	

Key subthemes identified in the analysis of this Twitter conversation included ‘PE Practices’(n = 3), ‘Team/Project Impact’ (n = 16), ‘Personal Impact’(n = 27), ‘Exclusion’(n = 23), ‘Inclusion’(n = 8), ‘Respect’(n = 4), ‘Disrespect’(n = 78), ‘Promotion’(n = 88), ‘Support’(n = 29). Most tweets illuminated shortcomings of PE procedures and guidelines and clustered around the original hashtag, ##HowNotToDoPatientEngagement. Additional tweets that highlighted positive experiences and examples of PE evolved into a parallel conversation around the hashtag, #HowToDoPatientEngagement.

**Fig. 2:**
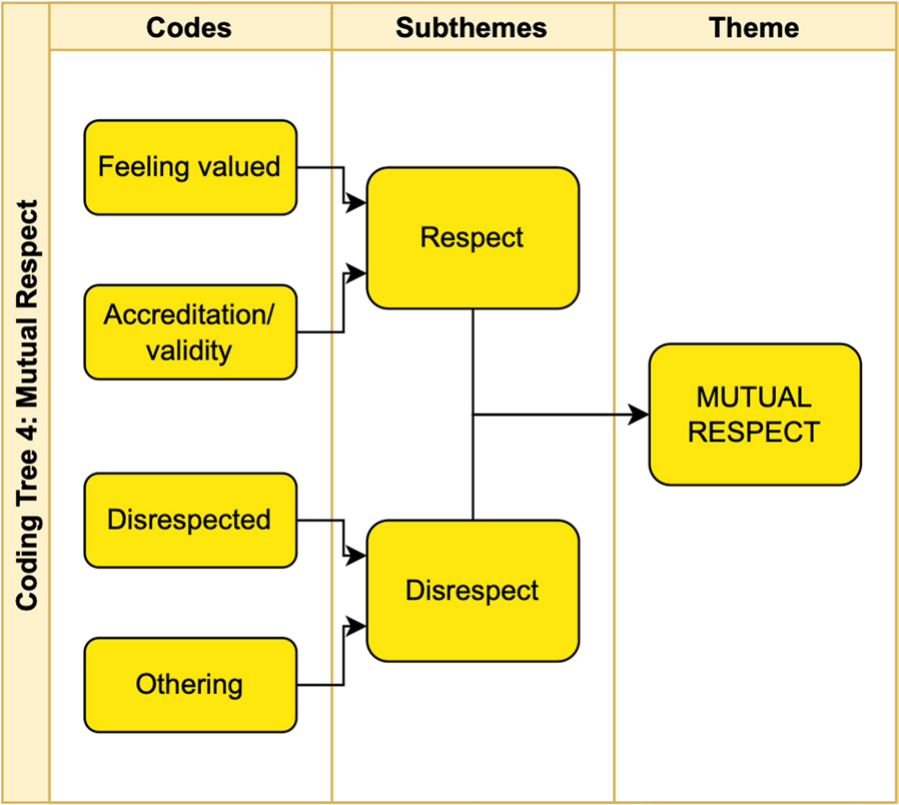
Sample coding tree for overall theme of ‘Mutual Respect’

While conducting our analysis we noted that even though most tweets critiqued current experiences with PE practices, the intentions were aimed at guiding the practice to evolve and improve, as to make these experiences better for future patient partners. Therefore, we grouped each negative theme with their corresponding positive counterpart (e.g., Exclusion/Inclusion; Respect/Disrespect). The emerging thematic groupings from hashtag subthemes were grouped into five main themes; ‘Mutual Respect’ (n = 82), ‘Co-build’ (n = 3), ‘Inclusiveness’ (n = 31), ‘Support’ (n = 117), and ‘Impact’ (n = 43). As qualitative researchers and patient partners conducting this study, similarities between the content analysis and the SPOR PE Framework were drawn. Study results were then used to validate this framework, and address components of the framework that could be further contextualised through the analysis of this Twitter conversation. The validated categories of the SPOR PE framework, plus emergent themes from our analysis, subthemes and illustrative quotes are shown in Table 3 and a sample coding tree is shown in Fig. 2.

**Table 3:** Themes, subthemes and illustrative quotes from tweet text

SPOR framework theme	Corresponding theme/ subthemes and illustrative quotes (contributor tweets)	Tweet count
muTUal respect	Mutual respect	82
	Subtheme: Respect	4
	Tweets in this subtheme present the action of mutual respect between researchers and patient partners, regardless of title or status	
	Subtheme: Disrespect	78
	Tweets in this subtheme highlighted examples of PE practices that made patient partners feel looked down upon, or facilitated differential treatment due to patient partner status. Tweets differentiate from ‘Exclusion’ subtheme, since actions we not exclusionary, however, posters felt undervalued	
	*I have been told that patients do not know enough and should stay out of it*	
Support	Community	117
	Subtheme: Promotion	88
	Inviting twitter followers to join the discussion on #HNTDPE and using interest in the hashtag to promote interest in patient engagement	
	*In Ontario, there is an active twitter discussion on #hownottodopatientengagement. It’s worth checking out. #ICIC18 @TheChangeFdn*	
	Subtheme: Support	29
	This subtheme seeks out advice from practitioners and patient partners, acknowledging patient partner leadership and sharing what they have learned from people with lived experience as patient partners. This subtheme highlighted the importance of peers and community building as a means of supporting PP	
	*We hope that you can make it — we can learn so much from you about #HowToDoPatientEngagement!!*	
Inclusivness	Inclusivity	31
	Subtheme: Exclusion	23
	The tweets in the subtheme consisted of codes relating to compensation and accessibility, were mainly negative, and regularly commented on the shortcomings of the current compensation of patient partners or barriers to partnership. Many users commented on specific experiences in order to justify a lack of appropriate payment in return for their expertise	
	*When a SPOR-funded entity doesn’t have a patient partner compensation policy… aka they aren’t compensating patient partners… I’ve started declining requests that don’t acknowledge our time & expertise*	
	Subtheme: Inclusion	8
	The inclusion subtheme shared resources developed by patient partners on the importance of compensation	
Co-build	Co-build	3
	Subtheme: PE practices	3
	This subtheme emphasizes positive PE practices through the dissemination of PE resources for existing standards in patient oriented research	
	*On March 20 the @OfficialNIHR, in conjunction with a host of U.K. associates, released a set of standards and indicators for public involvement in research, found here: *https://t.co/OK34P6MYUN* Another excellent resource for anyone interested in #HowToDoPatientEngagement *https://t.co/ngYor2bqWI	
N/A	Impact	43
	Subtheme: Impact as a value (see also Fig. 4)	27
	PP’s feelings of both the value of their contribution and feeling of fulfilment or lack thereof. Many of these tweets contained calls to action by PPs to recognize the current tokenistic treatment of patient partners that made them feel unvalued or used only to advantage the research team	
	*I see we are in the ‘academics use patients’ words for their own glory’ stage of patient engagement*	
	*Building on the experience of patients and families to improve care for everyone. Inspiring. #patientexperience #patientsafety #patientleadership #howtodopatientengagement So pleased for this mom that she had this opportunity and was supported to speak up*	
	Subtheme: Impact as an outcome	16
	This subtheme emphasizes PE outcomes that leave a positive impact on the patient partner and the project	
	*What would your response be if your name was attached to a report that you were not consulted on. Your name was only added to say that a Patient voice is there, in name only*	
	*What matters to me—not being turned into an infographic. What’s the purpose of this, and who’s the audience?*

## Interpretation

**Fig. 3:**
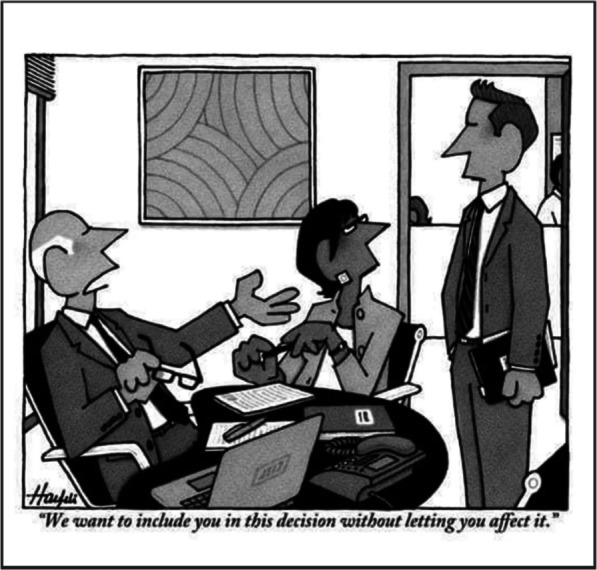
#HowNotToDoPatientEngagement – “How I feel at times.”

Current PE guidelines and best practices have been shaped by Canada’s Strategy for Patient-Oriented Research (a.k.a. SPOR), through the formation of their widely recognized SPOR Patient Engagement Framework. This framework was developed to act as a guide to appropriately involve patient partners in the design and conduct of health research projects [2]. However, it has been nearly a decade since any adjustments and/or advancements to this framework have been made [11]. The four guiding principles described in the SPOR Patient Engagement Framework are Co-building, Inclusiveness, Mutual Respect and Support. The five themes identified in our study align with the SPOR Guiding Principles, apart from a new emergent theme, ‘Impact’. In total, the thematic category, Impact, comprised 16% of the total tweets analysed and emphasizes the importance of impact to patient partners drawn from their own positive or negative experiences of PE. Based on our analysis, we define “Impact in patient engagement” as a positive outcome for the patient partner and the broader healthcare community that is a result of purposeful and intentional efforts to engage with patients for impact. Our study highlights the crucial inclusion of ‘impact’ as a core value that creates space for impact to become a defined outcome of patient partnership. Impact thus, becomes both a value-driven principle, that with intentionality can lead to better impact in patient-engaged outcomes. The academic literature on PE has grown dramatically since the SPOR Patient Engagement Framework was launched. This literature includes new evaluation tools and quality indices, which increasingly recognize that the processes of patient engagement are as important as the outcome of engagement [11, 12]. These tie tightly with our subthemes of impact as a process ‘value’ and impact as an ‘outcome’ (Table 3 and Fig. 3).

### The Engaging with Purpose Patient Engagement Framework

**Fig. 4:**
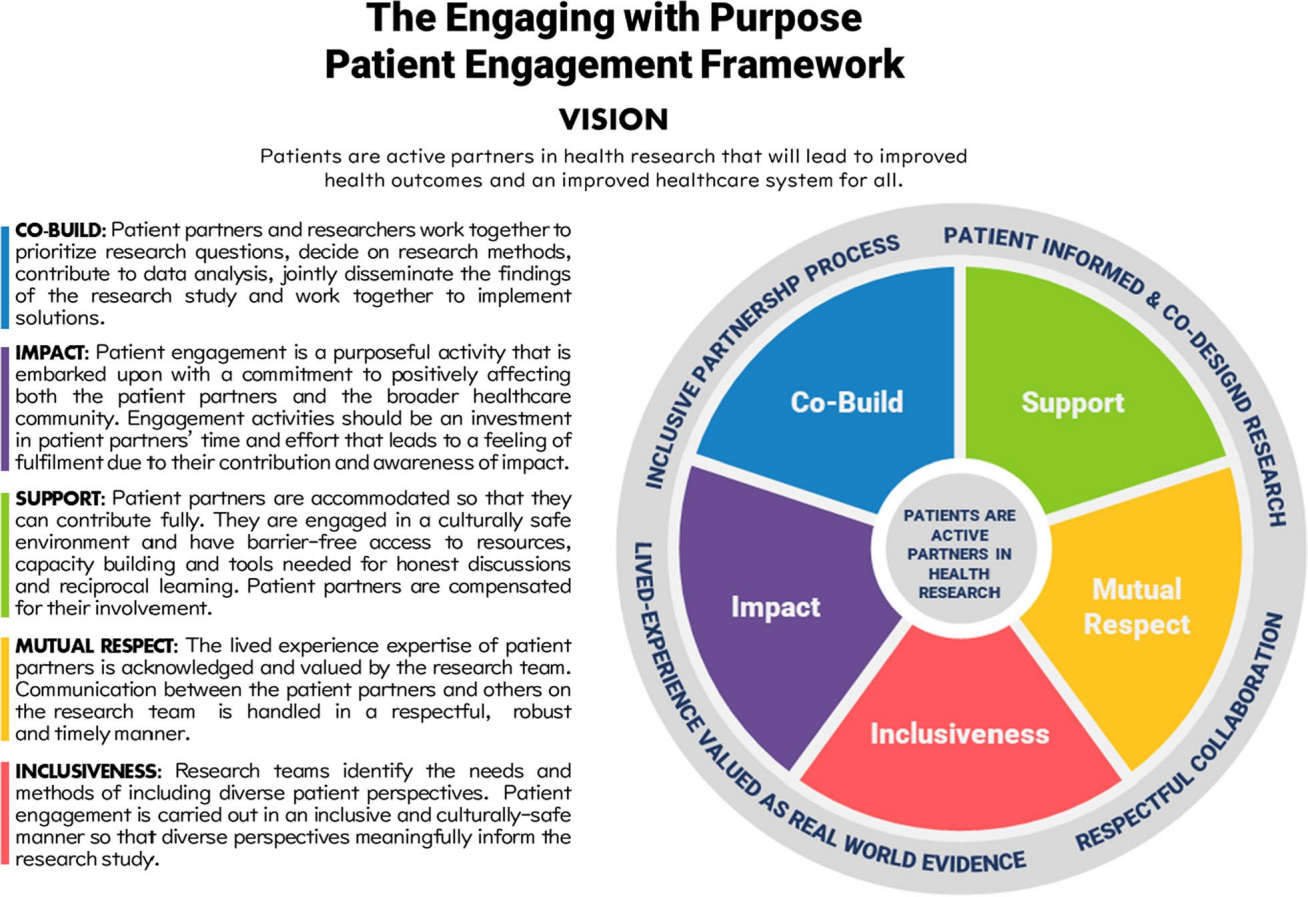
The Engaging with Purpose Patient Engagement Framework

The overarching message reflected in the hashtag, ‘#HowNotToDoPatientEngagement’ was the importance of proactively considering how one’s PE practices impact patients at a personal, project and overall health outcome level. From the thematic analysis, it is reinforced that patient partners want to know how their time and input has influenced project decisions and outcomes, this consideration seems to make the process of PE meaningful and drives value to patient partners. The Engaging with Purpose Patient Engagement Framework aims at purposeful engagement, with a goal of intentionally creating positive impact on both patient outcomes overall, while also immediately enhancing the experiences of patient partners. This is necessary to sustain and grow the practice of PE in a meaningful way. Our revised framework reflects the evolution in patient engagement practices over the past decade and we have co-built it based on the analysis of the unfiltered reflections of patient partners and practitioners who have engaged in partnerships. In the Engaging for Purpose Patient Engagement Framework, we describe guiding principles to promote intentional thinking, planning and action so that we are purposefully striving for positive impact (Fig. 4). In addition, we commit to evolving this framework as we continue to listen to patient partners and those practicing patient engagement on an ongoing basis.

### Limitations

Due to the data collection method used in this study, there are three main limitations that may have an effect on the interpretations of the study data. First, since this study was conducted via the collection of perspectives on Twitter specifically, the resulting analysis only speaks to a subset of online community members who use this social media platform. To mitigate this limitation, future research collecting user data across multiple social media and blogging platforms could be used to address a more expansive view of perspectives. Second, the API (Tweet Binder) was programmed to collect tweets with any combination of the hashtags #HNTDPE and #HTDPE. However, this search did not include tweet replies that did not use these hashtags, or tweets with variations of these hashtags that were a part of this dialogue. Therefore, some content may have been missed in the data analyses, effecting the frequency of recurring codes. Lastly, the data used to conduct qualitative analysis in this study came from self-reported data in the form of tweets. Often this reporting is retrospective in nature and therefore leaves room for recall bias of contributors when posting content.

## Conclusion

Patient engagement practices have come a long way in the past decade, due to the efforts of the CHIR’s Strategy for Patient Oriented Research and contributions of many patient partners and practitioners who have collaborated to improve health outcomes for patients across Canada. However, it is not often that we are offered an honest unfiltered measure of general patient engagement sentiment. The opportunity to analyze a Twitter conversation around #HowToDoPatientEngagement and #HowNotToDoPatientEngagement, is a unique one that provides insights on how to move the practice forward. Our analysis of this Twitter conversation found that the practice of patient engagement is rooted in the desire to make a difference and be treated with respect when doing so. Patient partners look to peers and others with lived experience in partnering to validate their thinking and move towards improving the practice overall. This may be the reason that the hashtags gained traction, and many contributed to it. Noting the importance of community building, impact and the desire for improvement, we build on the SPOR PE framework to propose an Engaging for Purpose Patient Engagement Framework, that considers impact, respect, support, collaboration and inclusivity from the perspective of those with lived experience in patient partnerships.

## Data Availability

The datasets analysed during the current study are available from the corresponding author on reasonable request.
